# Supplementary material for: Environmental conditions limit attractiveness of a complex sexual signal in the túngara frog
Source: Nat Commun. 2017 Dec 1;8:1891. doi: 10.1038/s41467-017-02067-1 (PMC5709490; doi:10.1038/s41467-017-02067-1)
Supplement: Supplementary file 1 — Supplementary Information [file 41467_2017_2067_MOESM1_ESM.pdf]

Supplementary information

Supplementary Table 1. Results from a generalised mixed model assessing the effect of sound playback and water depth on male call behaviour (n = 20 males).

Dependent variable	fixed effects	parameter estimate	s.e.	χ^2	df	p-value
**Vocal sac width**	Intercept	19.83	0.69			
	Water Depth	1.81	0.22	62.09	1	< 0.001
	Sound playback			13.09	2	< 0.001
	(chorus)	1.10	0.36			
	(single call)	1.23	0.37			
**Body width**	Intercept	21.05	0.61			
	Water Depth	1.25	0.18	48.50	1	< 0.001
	Sound playback			8.41	2	= 0.015
	(chorus)	0.52	0.28			
	(single call)	0.85	0.29			
**Call rate**	Intercept	0.09	0.04			
	Water Depth	0.18	0.02	34.81	1	< 0.001
	Sound playback			205.6	2	< 0.001
	(chorus)	0.19	0.01			
	(single call)	0.05	0.01			
**Call complexity**	Intercept	-0.19	0.04			
	Water Depth	0.20	0.03	26.18	1	< 0.001
	Sound playback			347.9	2	< 0.001
	(chorus)	0.40	0.02			

	(single call)	0.27	0.02			
**Peak amplitude whine**	Intercept	0.079	0.016			
	Water Depth	0.052	0.007	25.85	1	< 0.001
	Sound playback			49.53	2	< 0.001
	(chorus)	0.031	0.004			
	(single call)	0.017	0.004			
**Chuck-to-whine-ratio**	Intercept	0.36	0.15			
	Water Depth	0.28	0.06	13.75	1	< 0.001
	Sound playback			43.28	2	< 0.001
	(chorus)	0.33	0.07			
	(single call)	0.49	0.07			

Supplementary Table 2. Differences in body vibrations recorded from the body wall or vocal sac. We measured RMS amplitude of the whine and chuck part separately and calculated the ratio between them for both body parts (value of < 0 indicates relative stronger vibrations of the chuck recorded from the vocal sac and when compared to the body wall). We also calculated the ratio in the low- and high-frequency ranges corresponding to the dominant frequency ranges in airborne component of whine and chuck respectively (see also Fig. 1).

**Dependent variable**	**fixed effects**	**parameter estimate**	**s.e.**	χ^2	**df**	**p-value**
Overall W-C ratio	Intercept	0.10	0.12			
	Vocal sac	-0.26	0.06	15.11	1	< 0.001
Ratio low-frequency	Intercept	1.55	0.58			
	Vocal sac	-2.83	0.80	67.42	1	< 0.001

Ratio high-frequency	Intercept	1.85	0.33			
	Vocal sac	-1.87	0.44	40.01	1	< 0.001

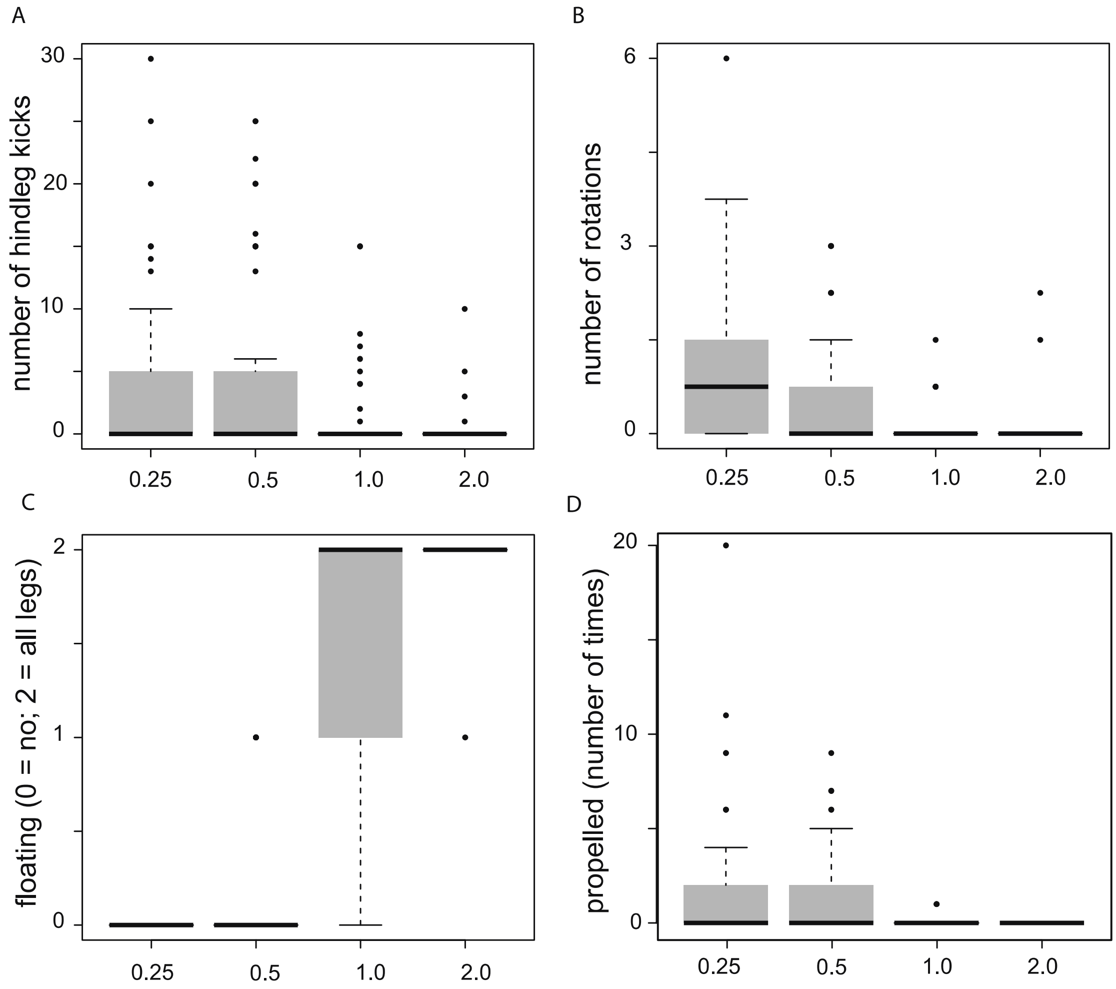

Supplementary Figure 1. Water depth influences non-calling behaviour. A) Effect of water depth on the ability for males to float. Floating was scored as either all legs touching the floor (0), at least one pair of legs touching the floor (1) or no legs touching the floor (2). In shallow water (0.25-0.5 cm) males were hardly able to float. Furthermore, males would rotate more often (**B**) and would kick their hind legs more often when confronted with shallow water conditions (**C**). Males would occasionally shoot forward during calling, a behaviour we termed propelling and which increased

with decreasing water levels (**D**). As a consequence of this propelling behaviour, males would have to reposition before they could regain calling.
