# Supplementary material for: Environmental conditions limit attractiveness of a complex sexual signal in the túngara frog
Source: Nat Commun. 2017 Dec 1;8:1891. doi: 10.1038/s41467-017-02067-1 (PMC5709490; doi:10.1038/s41467-017-02067-1)
Supplement: Supplementary file 3 — Description of Additional Supplementary Files [file 41467_2017_2067_MOESM3_ESM.pdf]

Description of Supplementary Files

File Name: Supplementary Movie 1
Description: Examples of non-acoustic behaviour.

File Name: Supplementary Movie 2
Description: Effect of water depth treatment on male calling behaviour.

File Name: Supplementary Movie 3
Description: Slow-motion video of calling male frogs.
